# Quality, scope and reporting standards of randomised controlled trials in Irish Health Research: an observational study

**DOI:** 10.1186/s13063-020-04396-x

**Published:** 2020-06-08

**Authors:** Barbara Clyne, Fiona Boland, Norah Murphy, Edel Murphy, Frank Moriarty, Alan Barry, Emma Wallace, Tatyana Devine, Susan M. Smith, Declan Devane, Andrew Murphy, Tom Fahey

**Affiliations:** 1 HRB Centre for Primary Care Research, Department of General Practice, Royal College of Surgeons in Ireland, 123 St Stephens Green, Dublin 2, Ireland; 2 Public and Patient Involvement (PPI) Ignite, NUI Galway, Galway, Ireland; 3 HRB-Trials Methodology Research Network, School of Nursing & Midwifery, NUI Galway, Galway, Ireland; 4 HRB Primary Care Clinical Trials Network Ireland, Department of General Practice, NUI Galway, Galway, Ireland

## Abstract

**Background:**

Despite efforts to improve the accuracy and transparency of the design, conduct, and reporting of randomised controlled trials (RCTs), deficiencies remain. Such deficiencies contribute to significant, avoidable waste of health research investment and impede reproducibility. This study aimed to synthesise and critically analyse changes over time in the conduct and reporting of internationally published evidence on patient and/or population health-oriented RCTs conducted in one country.

**Methods:**

This observational study drew on systematic review methods. We searched six databases for published RCTs (database inception to December 2018) where ≥ 80% of participants were recruited in the Republic of Ireland. RCTs of interventions targeted at patients, providers and/or policy makers intended to improve health, healthcare or health research were included. For each study, screening, data extraction and methodological quality appraisal were conducted by one member of the author team.

**Results:**

From 17,560 titles and abstracts, 752 unique RCTs were published in 745 papers between 1968 and 2018, with a steady year-on-year increase since 1968. The number of participants was in the range of 2–8628. The majority were parallel design (86%) and classified as treatment evaluation. Of the 418 RCTs published since the introduction of mandatory clinical trial registration by the International Committee of Medical Journal Editors in 2005, 32% (*n* = 134) provided a trial registration number. This increased to 47% when taking studies published between 2013 and 2018 (*n* = 232). Since the 1996 publication of the CONSORT statement, 16% of included RCTs made specific reference to a standardised reporting guideline and this increased to 31% for more recent studies published between 2013 and 2018. Overall, 7% (*n* = 53) of studies referred to a published study protocol, increasing to 20% for studies published between 2013 and 2018.

**Conclusion:**

Evidence from this single-country study of RCTs published in the international literature suggests that both the number overall, the number registered and the number referencing reporting guidelines have increased steadily over time. Despite widespread endorsement of reporting standards, reporting of RCTs remains suboptimal in domains such as compliance with the CONSORT statement and prospective trial registration. Researchers, funders and journal editors, nationally and internationally, should continue to focus on improving reporting and examining avoidable waste of health research investment.

## Background

Randomised controlled trials (RCTs), when appropriately designed, conducted and reported, represent the gold standard in evaluating the effectiveness of healthcare interventions and have traditionally been considered the backbone of evidence-based medicine [1, 2]. The findings of RCTs should be published, rigorously conducted and clearly reported to inform decision making.

The number of RCTs conducted and reported has continually increased over time and it is difficult to quantify the exact volume of this research activity [3]. The extent to which published RCTs reflect the efficacy of interventions, however, depends on the completeness and accuracy of published results. Two important initiatives have emphasised the need to increase the accuracy and transparency with respect to the performance and reporting. In 2005, the International Committee of Medical Journal Editors (ICMJE) introduced mandatory trial registration guidelines and member journals require prospective registration of RCTs before patient enrolment as a condition of publication [4]. The Consolidated Standards of Reporting in Trials (CONSORT) reporting guideline was developed and published in 1996 to improve the accuracy and transparency of reports of RCTs [1]. Both initiatives aim to increase the adequacy of reporting of important aspects of how trials are designed, analysed and interpreted to overcome issues such as outcome switching or publication bias. Despite these and other efforts to improve the accuracy and transparency of the design, conduct, analysis and interpretation of RCTs, deficiencies and lack of adequate reporting continue [1]. A large systematic review updated in 2012 showed that, despite improvements in the completeness of reporting for 22 of 25 CONSORT checklist items, there are still major reporting deficiencies in some areas [5]. Subsequent reviews and research have confirmed that reporting remains suboptimal in areas such as sample size calculations and consistency between pre-specified and reported outcomes [6–9]. Adherence to the CONSORT statement for Abstracts is also variable and incomplete [10]. Adherence to the ICMJE’s prospective registration policy also remains suboptimal, with high-impact journals frequently publishing unregistered trials and trials registered after potential observation of primary outcome data [11]. Such deficiencies contribute to significant and avoidable waste of health research investment and impede reproducibility [12].

It has been suggested that research-active organisations and companies should routinely audit their activity with regard to the registration and publication of RCTs [13, 14]. Over the last 20 years, there has been significant investment in improving health research in Ireland, highlighting a commitment to developing research capacity [15, 16]. There is also a shift away from funding biomedical research (broadly focused on the investigation of the biologic process and the causes of disease through careful experimentation, observation, laboratory work, analysis and testing) towards population health (research with the goal of improving the health of the population through investigation of social, cultural, environmental, occupational and economic factors or through the identification of effective interventions) and health services research (research with the goal of improving the efficiency and effectiveness of health professionals and the healthcare system) by some funding bodies such as the Health Research Board of Ireland [17].

Current research indicates that substantial amounts of publicly funded RCTs are not published or available to inform future research and practice [18]. In this context, it is important to evaluate locally generated and published research and use this to improve standards and to contextualise the evidence that informs local healthcare and health policy. Th aim of the present study was to synthesise and critically analyse the changes in the reporting of published evidence from patient or population health-oriented RCTs conducted in one country, the Republic of Ireland, over time.

## Methods

This observational study drew on systematic review methods to identify, appraise and synthesise RCTs. As we were not attempting to determine the effectiveness of any one particular intervention, a full systematic review was not deemed necessary. We used a systematic review approach to conducting the literature search but literature screening, data extraction and quality appraisal were not conducted in duplicate, as would be standard practice in a systematic review.

### Data sources and search strategy

A literature search was performed including PubMed, Embase, Scopus, CINAHL, PsychINFO and the Cochrane Central Register of Controlled Trials, from database inception to December 2018. Within MEDLINE, we used the methodology filter for RCTs, ‘Randomized Controlled Trial’ (Publication Type), which has a sensitivity for retrieving RCTs of approximately 94% [19, 20]. Combinations of key words and MeSH terms were used to search the other databases. The search strategy was developed with the support of a Health Sciences Librarian with expertise in systematic review searching (Additional file 1). No language restrictions were applied.

### Study selection

Full-text studies were included if they met the following inclusion criteria:

1. Population: all studies conducted on human subjects of all age groups in an Irish healthcare setting (hospital and community) or where ≥ 80% of participants were recruited in the Republic of Ireland. International studies where ≥ 80% of participants were recruited in Ireland were also included.
2. Intervention: Any intervention targeted at patients, providers and/or policy makers intended to improve health, healthcare or health research (including biomedical research and population health and health services research).
3. Comparators: no limitation on comparators.
4. Primary outcome: any health outcome focused on patient/population health.
5. Study design: described by authors as a RCT of any design (e.g. parallel, cluster, factorial, cross-over, stepped wedge), with two or more groups. However, author-described RCTs which did not state explicitly that random allocation was employed were managed by consensus and studies that stated information to the contrary were excluded, e.g. authors stating the paper as a RCT but describing recruitment as consecutive. Clinical trial phases II and above were also included, so long as they were randomised.

Initially identified study titles were divided between authors and assessed in relation to the inclusion criteria by reading titles and abstracts. Potentially eligible studies were read fully and their suitability for inclusion determined individually by author team members. Full-text papers were obtained from a combination of institutional library access, online profiles (such as Researchgate) and by contacting authors. Multicentre, international studies where there was one or more Irish centre but < 80% of participants recruited in Ireland were recorded, but are not included in the analysis presented here.

### Data extraction

Included full texts were divided between authors and reviewers extracted data from the included studies using a pre-established data extraction form. This was not done in duplicate. The reviewers extracted the information on study characteristics, participant demographics, study documentation and methodological quality from each study, including:

1. study characteristics: author, year of publication, study title, journal, study design as reported by the authors, objectives, inclusion criteria, clinical domain or condition as reported by the authors, clinical setting, composition of intervention/control groups, study dates and duration, number of outcomes, results for primary outcome (positive or negative, significant or non-significant), sample size, funding source.
2. documentation of reporting quality: registration number, protocol publication (yes or no), standardised reporting guideline explicitly used (yes or no).
3. methodological quality: Cochrane domains (see below).

The Health Research Classification System (HRCS) [21], developed by The UK Clinical Research Collaboration (UKCRC), was used to categorise research activity. The specific interventions were further classified by the primary disease focus (coded using International Classification of Diseases, 10th revision [ICD-10]), the primary procedures (using the Australian Classification of Health Interventions [ACHI]), the primary behavioural intervention (provider or patients) or the primary service delivery intervention.

### Methodological quality

One reviewer individually assessed the risk of bias of each RCT using the Cochrane Collaboration’s risk of bias tool including the standard domains of sequence generation, allocation concealment, blinding of participants, blinding of outcome assessors, incomplete outcome data, selective reporting and other bias.

### Data synthesis

A narrative synthesis of included studies is presented. Descriptive statistics are used to summarise trends over time in terms of the quantity and reporting quality of included RCTs across all years and a subset of the most recently published studies. A pragmatic cut-off of studies published between 2013 and 2018 was used as the most recently published.

## Results

**Fig. 1:**
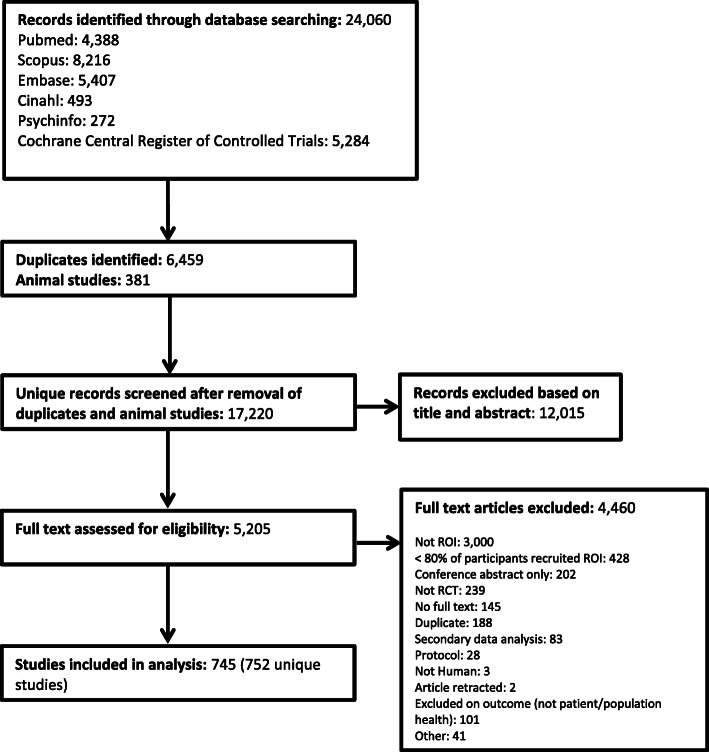
Flow diagram of studies screened

**Fig. 2:**
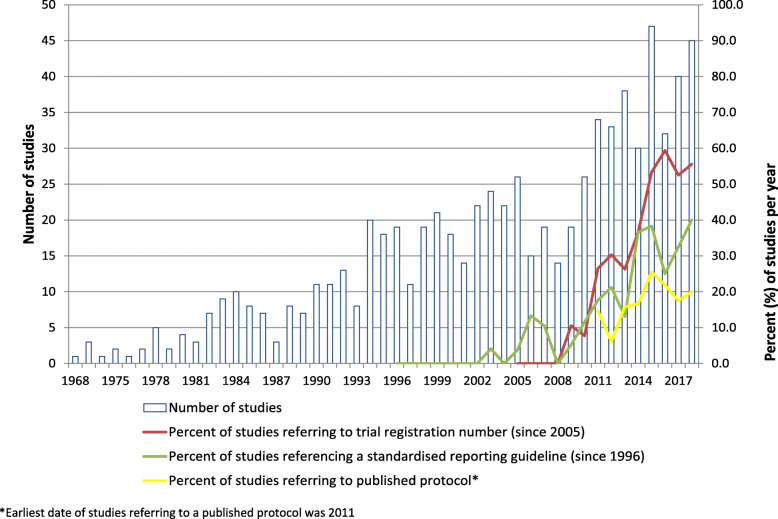
Number of studies published and proportion meeting reporting standards per year

We screened 17,220 titles and abstracts and identified 5205 studies for full-text review. Of these, 752 unique RCTs from 745 papers (some papers reported greater than one RCT) published between 1968 and 2018 were included (Fig. 1). The number of studies published per year has steadily been increasing since 1968 (Fig. 2). The majority of papers were published in international journals, with 2% publishing in an Irish specific journal (e.g. *Irish Medical Journal*).

### Characteristics of included studies

#### Study design, setting and population

As shown in Table 1, the majority of studies were of parallel design (*n* = 643, 85%) and were most frequently conducted in the hospital, inpatient setting (*n* = 314, 42%). Less than 10% (*n* = 73) were conducted in primary care. The majority of studies were conducted with adult populations (*n* = 549, 73%), with older people (≥ 65 years, *n* = 43) and pregnant women (*n* = 47) being the specified population in 6% of studies each. The number of participants included in each RCT was in the range of 2–8628 (median 60, mean 142).

**Table 1:** Study characteristics

Characteristic^**a**^	n (752)	%
**RCT design**		
Parallel	643	85.5
Crossover	76	10.1
Cluster	23	3.1
Other	10	1.3
**Clinical setting**		
Hospital (inpatient)	314	41.8
Ambulatory (outpatient)	276	36.7
Primary care	70	9.3
Other	92	12.2
**Population**		
Adults	549	73.0
Pregnant women	47	6.3
Older people (≥ 65 years)	46	6.1
Children (≤ 18 years)	43	5.7
Infants (author defined)	16	2.1
Other	41	5.5
Not stated	10	1.3
**Comparator**		
Alternative intervention	298	39.6
Usual care	270	35.9
Placebo	160	21.3
Other	24	3.2
**Funding source**		
Not stated	412	54.8
Government body/agency	103	13.7
Industry	89	11.8
Multiple funders	47	6.3
Charity	40	5.3
University	14	1.9
No funding	3	0.4
Other	44	5.9

^a^ All categories are mutually exclusive

#### Research activity and interventions

An analysis by research activity (using HRCS categories) showed the majority of research was in the domain of treatment evaluation, with the top three areas of activity focused on pharmaceuticals (*n* = 556, 47%), physical activity (*n* = 88, 12%) and psychological and behavioural interventions (*n* = 75, 10%) (Table 2). The main comparators (Table 1) were an alternative intervention (*n* = 298, 40%), usual care (*n* = 270, 36%) or placebo (*n* = 160, 21%).

**Table 2:** Health Research Classification System (HRCS) category research activity

HRCS category	n (752)	%
**Aetiology (*n*** **= 2)**		
2.1 Biological and endogenous factors	1	0.13
2.3 Psychological, social and economic factors	1	0.13
**Prevention of disease and conditions, and promotion of wellbeing (*n*** **= 6)**		
3.2 Interventions to alter physical and biological environmental risks	5	0.66
3.3 Nutrition and chemoprevention	1	0.13
**Detection, screening and diagnosis (*n*** **= 12)**		
4.2 Evaluation of markers and technologies	10	1.33
4.4 Population screening	2	0.27
**Evaluation of treatments and therapeutic interventions (*n*** **= 701)**		
6.1 Pharmaceuticals	356	47.34
6.3 Medical devices	71	9.44
6.4 Surgery	69	9.18
6.5 Radiotherapy and other non-invasive therapies	21	2.79
6.6 Psychological and behavioural	75	9.97
6.7 Physical	88	11.70
6.8 Complementary	3	0.40
6.9 Resources and infrastructure (treatment evaluation)	18	2.39
**Health and social care services research (*n*** **= 31)**		
8.1 Organisation and delivery of services	31	4.12

Over half (*n* = 401, 53%) of all studies focused on specific conditions with diseases of the circulatory system being the most frequently studied (*n* = 52, 13%). The conduct of procedures (e.g. hip surgery) were the primary focus in 34% (*n* = 253) of studies with non-invasive, cognitive and other interventions being most common in that category (*n* = 415, 45%). The top five conditions and procedures are presented in Table 3. The remaining studies were classified as behavioural intervention (provider or patients) or the primary service delivery interventions.

**Table 3:** Top five diseases or conditions and procedures

**Disease or conditions**	**n (401)**	**%**
Diseases of the circulatory system	52	13
Mental and behavioural disorders	47	11.7
Diseases of nervous system	40	10
Diseases of the digestive system	37	9.2
Diseases of the musculoskeletal system	22	5.5
**Procedures**	**n (253)**	**%**
Non-invasive, cognitive and other interventions, not elsewhere classified	115	45.5
Obstetric procedures	29	12.5
Procedures on musculoskeletal system	21	8.3
Procedures on digestive system	16	6.3
Procedures on cardiovascular system	15	5.9

#### Funding

Of the 752 RCTs, 412 (55%) did not report their sources of funding. Government bodies were cited by 14% (*n* = 103), industry funding was cited by 12% (*n* = 89), with the remaining studies funded by charities, universities and other funders. Taking studies published between 2013 and 2018 (*n* = 232) as a subset of the most recently published, 35% (*n* = 81) did not report their sources of funding, government bodies were cited by 28% (*n* = 65), industry funding was cited by 5% (*n* = 12), with the remaining studies funded by charities, universities and other funders.

### Reporting quality

Of the 418 RCTs published since the ICMJE introduced mandatory clinical trial registration in 2005, 32% (*n* = 134 published between 2005 and 2018) provided a trial registration number although year-on-year increases were observed (Fig. 2). Taking studies published between 2013 and 2018 (*n* = 232) as a subset of the most recently published, this figure rose to 47% (*n* = 109) providing a trial registration number. Overall, 7% (*n* = 53) of studies referred to a published study protocol, with 20% (*n* = 47) of studies published between 2013 and 2018 referring to a published study protocol. Sixteen percent of RCTs published between 1996 (the CONSORT statement original publication year) and 2018 made specific reference to a standardised reporting guideline within the publication. Year-on-year increases were observed here also, with 31% (*n* = 72) of studies published between 2013 and 2018 making specific reference to a standardised reporting guideline (Fig. 2). Only 304 (40%) RCTs mentioned a sample size calculation, with 273 achieving the required sample size. Year-on-year increases were observed here also with 62% (*n* = 143) of studies published between 2013 and 2018 referring to a sample size calculation. Overall, 79% (*n* = 594) of studies reported having obtained approval by an ethics committee.

### Methodological quality

**Fig. 3:**
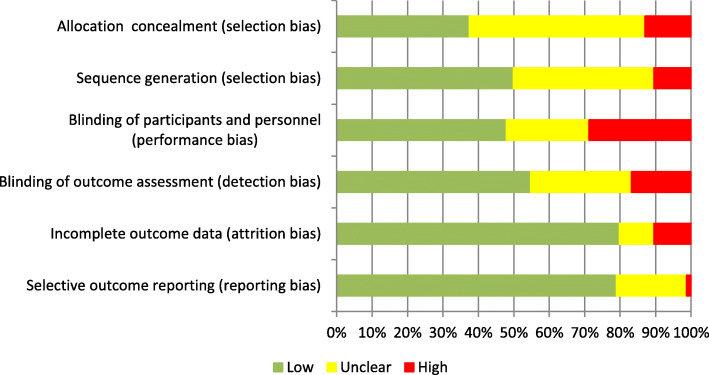
Risk of bias graph

As summarised in Fig. 3, the risk of bias was low in the majority of studies for attrition bias (*n* = 594, 79%), reporting bias (*n* = 594, 79%) and detection bias (*n* = 414, 55%). Risk of bias was high or unclear in the majority of studies for performance bias (*n* = 392, 52%), allocation concealment (*n* = 467, 62%) and sequence generation (*n* = 376, 50%).

## Discussion

This study identified 752 unique RCTs published internationally across 745 papers between 1968 and 2018. Evidence from this single-country, observational study suggests that both the number and the reporting quality of RCT articles have increased steadily over time. However, reporting of RCTs remains suboptimal, with only 32% of included RCTs published since 2005 providing a trial registration and 16% of included RCTs published since 1996 making specific reference to a standardised reporting guideline; therefore, areas for improvement in a number of domains were identified.

Prospective registration of RCTs is critical for promoting transparency and integrity in research by discouraging undeclared protocol deviations or outcome switching which increase the likelihood that reported effects have arisen through bias, chance or are exaggerated. The RCTs identified in this study were particularly poor in adhering to the ICJME’s prospective registration policy, with only 32% of the RCTs published since 2005, the year ICMJE introduced mandatory clinical trial registration, providing a trial registration number and < 10% of studies overall referring to a published study protocol. This suggests that authors are either not registering trials or not reporting trial registration numbers, and journal editors are either not checking trial registries for appropriate registration or have identified reasons for non-registration and chose to publish it without transparently providing any documentation of this decision-making process. This is not an issue specific to research in Ireland. Adherence to the ICMJE’s prospective registration policy has been identified as suboptimal internationally, with high-impact journals frequently publishing unregistered trials and trials registered after potential observation of primary outcome data [11, 22]. The RCTs identified in this study were also poor in referring to and adhering to several items the CONSORT statement. The COMPare (Centre for Evidence-Based Medicine Outcome Monitoring Project) study has recently highlighted, based on five high-impact journals endorsing CONSORT, that journals exhibit extensive breaches of the CONSORT guidance, particularly with respect to outcome reporting [9]. Our results confirm and extend this finding, given that we did not restrict by journal and the 752 RCTs were published across a range of journals and health research domains.

A particular area of poor reporting was study funding. The majority of studies included in this study did not report the source of funding within their manuscripts, although reporting in this area has improved over time. Most journals in which these papers were published, particularly for newer publications, require the reporting of the funding source. This reflects both a suboptimal compliance by authors with this requirement and poor implementation by journals internationally. The reporting of funding sources is necessary to allow adequate appraisal of primary research by individuals, those involved in evidence synthesis and decision makers. Funding sources may influence the reporting of research findings and the interpretation of results. Industry-funded trials are more likely to report favourable efficacy results and conclusions than those funded by other sources [23, 24]. Where RCTs do report funding, considerable variability in the reporting of funding source, amount and roles of funders has been identified and a standardised approach to reporting of funding information has been proposed [25]. However, given the poor compliance to reporting guidelines identified here and in the international literature [5, 26], it is unlikely that such standards would be adhered to. Use of reporting guidelines is largely determined by a combination of factors such as individual factors (e.g. having multiple reasons for use of reporting guidelines), the professional culture in which people work, environmental factors (e.g. policies of journals) and practical factors (e.g. having time to use reporting guidelines) [27]. Therefore, multifaceted interventions that target these factors are required to increase compliance [27].

### Implications

We would echo the COMPare project in their call for greater journal transparency in how they comply with CONSORT in terms of outcome reporting, with greater consistency in use of trial registries and changes to CONSORT’s mechanisms for enforcement [9]. It is also important to raise awareness in the peer-review process for the need to compare information recorded on a trial register to that reported in the manuscript as peer reviewers may not see this as their role, especially as editors may not clearly request the reviewer to assess this [28]. This may be time-consuming, so, alternatively, authors should be required to submit a copy of their registration as part of the journal submission process. Increased focus on improving peer review may be a mechanism to improve reporting quality. A number of journals have educational and training material available to improve peer review; however, training in the process is not mandatory. Given the increasing funding allocated each year to evaluate and develop medical treatments, understanding the patterns of publication of research findings among publicly funded research is important for public benefit, to identify areas of good and bad practice, reduce research waste and identify ways to better utilise public funding. Like journal editors, funders such as the Health Research Board (HRB) of Ireland and Science Foundation Ireland may have a role in enforcing greater transparency in complying with reporting guidelines and mandatory trial registrations.

### Limitations

We conducted an extensive search of the published literature across six electronic databases. However, this study has some limitations including the inherent difficulty in identifying RCTs from a specific geographical location. We encountered two main obstacles in this process. First, most databases do not easily facilitate the identification of the country of origin of the study or study population. We used combinations of key words and controlled vocabulary in the search string. We did not include search terms based on author affiliation as some databases limit this field to the first author affiliation only and we found variability in how authors report their affiliations (for example, it was not always clear if the affiliation was their current position or where the research was conducted). Second, in screening published papers, we found a large number of studies that gave no information at all as to where the study was conducted so we therefore excluded those studies. Third, we did not conduct a grey literature search or include forwards or backwards citation checking as part of our search, as this was not a full systematic review. Taken together, these limitations might have resulted in some omissions, underestimating the rate of publication. We would recommend that future publications are more specific about reporting where studies are conducted.

While this observational study drew on systematic review methods to identify, appraise and synthesis RCTs, it was not a full systematic review. Given the size and scope of the literature, the team did not conduct all screening and data extraction in duplicate which also might have induced some omissions of included studies and data errors. A full assessment of adherence to all the CONSORT statement items was not undertaken.

## Conclusion

Evidence from this single-country study suggests that both the number and the quality of RCT articles published internationally have increased steadily over time. Despite widespread endorsement of reporting standards, reporting of RCTs remains suboptimal in terms of adhering to several items the CONSORT statement and prospective trial registration, in keeping with international literature. Future research and journal editors should focus on improving reporting and examining avoidable waste of health research investment.

## Supplementary information

**Additional file 1.** Search string.

## Data Availability

Data have been uploaded to Zenodo 10.5281/zenodo.3754967.
