# Supplementary material for: Quality, scope and reporting standards of randomised controlled trials in Irish Health Research: an observational study
Source: Trials. 2020 Jun 8;21:494. doi: 10.1186/s13063-020-04396-x (PMC7278139; doi:10.1186/s13063-020-04396-x)
Supplement: Supplementary file 1 — Additional file 1. Search string. [file 13063_2020_4396_MOESM1_ESM.docx]

| **Search terms: PubMed** |
| --- |
| (((ireland) OR ireland[MeSH Terms])) AND (((((randomized controlled trial[Publication Type]) OR Controlled clinical trial[Publication Type]) OR ((control*[Title/Abstract]) AND trial[Title/Abstract])) OR ((clinical[Title/Abstract]) AND trial[Title/Abstract])) OR random allocation[Title/Abstract]) |

Additional file 1: Search string
